# *CDKN2A/B* Homozygous Deletions in Astrocytomas: A Literature Review

**DOI:** 10.3390/cimb45070335

**Published:** 2023-06-22

**Authors:** Alexander Yuile, Laveniya Satgunaseelan, Joe Q. Wei, Michael Rodriguez, Michael Back, Nick Pavlakis, Amanda Hudson, Marina Kastelan, Helen R. Wheeler, Adrian Lee

**Affiliations:** 1 Department of Medical Oncology, Royal North Shore Hospital, Sydney, NSW 2065, Australia; 2 Faculty of Medicine and Health, School of Medicine, University of Sydney, Camperdown Campus, Sydney, NSW 2000, Australia; 3 The Brain Cancer Group, North Shore Private Hospital, Sydney, NSW 2065, Australia; 4 Department of Neuropathology, Royal Prince Alfred Hospital, Sydney, NSW 2050, Australia; 5 Department of Pathology, Prince of Wales Hospital, Sydney, NSW 2065, Australia; 6 Department of Radiation Oncology, Royal North Shore Hospital, Sydney, NSW 2065, Australia

**Keywords:** *CDKN2A/B* alterations, *CDKN2A/B* homozygous deletions, *CDKN2A/B* heterozygous deletions, grade 4 astrocytomas, IDH mutant glioblastomas, radiotherapy, temozolomide

## Abstract

Genomic alterations of *CDKN2A* and *CDKN2B* in astrocytomas have been an evolving area of study for decades. Most recently, there has been considerable interest in the effect of *CDKN2A* and/or *CDKN2B* (*CDKN2A/B*) homozygous deletions (HD) on the prognosis of *isocitrate dehydrogenase* (*IDH*)-mutant astrocytomas. This is highlighted by the adoption of *CDKN2A/B* HD as an essential criterion for astrocytoma and *IDH*-mutant central nervous system (CNS) WHO grade 4 in the fifth edition of the World Health Organisation (WHO) Classification of Central Nervous System Tumours (2021). The *CDKN2A* and *CDKN2B* genes are located on the short arm of chromosome 9. *CDKN2A* encodes for two proteins, p14 and p16, and *CDKN2B* encodes for p15. These proteins regulate cell growth and angiogenesis. Interpreting the impact of *CDKN2A/B* alterations on astrocytoma prognosis is complicated by recent changes in tumour classification and a lack of uniform standards for testing *CDKN2A/B*. While the prognostic impact of *CDKN2A/B* HD is established, the role of different *CDKN2A/B* alterations—heterozygous deletions (HeD), point mutations, and promoter methylation—is less clear. Consequently, how these alternations should be incorporated into patient management remains controversial. To this end, we reviewed the literature on different *CDKN2A/B* alterations in *IDH*-mutant astrocytomas and their impact on diagnosis and management. We also provided a historical review of the changing impact of *CDKN2A/B* alterations as glioma classification has evolved over time. Through this historical context, we demonstrate that *CDKN2A/B* HD is an important negative prognostic marker in *IDH*-mutant astrocytomas; however, the historical data is challenging to interpret given changes in tumour classification over time, variation in the quality of evidence, and variations in the techniques used to identify *CDKN2A/B* deletions. Therefore, future prospective studies using uniform classification and detection techniques are required to improve the clinical interpretation of this molecular marker.

## 1. Introduction

**Figure 1:**
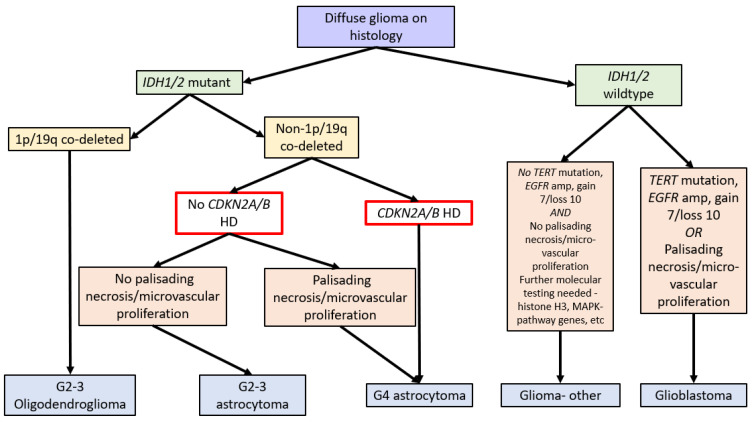
Flow diagram summarising the WHO Classification of CNS Tumours (fifth edition, 2021) [14] and the role of *CDKN2A/B* HD (red box). Note: gain 7/loss 10 refers to the gain of chromosome 7 and the loss of chromosome 10.

In 2016, the WHO Classification of Tumours of the Central Nervous System (revised 4th edition) incorporated *isocitrate dehydrogenase 1* and *2* (*IDH1/2*) mutation status into the classification of diffuse gliomas [1]. IDH1/2 mutations (point mutations in either IDH1 codon R132 or IDH2 codon R172) lead to IDH dysfunction, which converts alpha-ketoglutarate to R-2-hydroxygluterate, driving oncogenesis and global epigenetic changes [1,2,3,4]. The WHO Classification in 2016 further stratified diffuse IDH-mutant gliomas into oligodendrogliomas and astrocytomas based on the respective presence or absence of chromosome 1p and 19q co-deletions (see Figure 1) [1].

IDH-mutant astrocytomas account for 80% of WHO grades 2 to 3 and 5% of high-grade astrocytomas [5,6]. When compared to IDH-wildtype glioblastomas, patients with IDH-mutant astrocytomas are younger at diagnosis (30–40 years vs. over 50 years). In addition, IDH-mutant astrocytomas have a more favourable prognosis compared to IDH-wildtype glioblastomas, even in high-grade cases, with grade 4 IDH-mutant astrocytomas having a median overall survival (OS) of 31 months, compared to IDH-wildtype glioblastomas with a median OS of 13 months [5,6]. Unfortunately, a proportion of IDH-mutant astrocytomas have poor outcomes similar to those of IDH-wildtype glioblastomas [7]. 

CDKN2A/B HD are identified in approximately 22% of IDH-mutant astrocytomas [8] and are thought to lead to the loss of cell cycle control and promote cell proliferation [9]. Several retrospective studies have shown CDKN2A/B HD is associated with decreased survival among IDH-mutant astrocytomas [10,11,12,13]. A timeline of discovery and key developments in the understanding of CDKN2A/B deletions is presented in Table 1.

Due to the improved prognosis of tumours previously classified as IDH-mutant glioblastomas compared to IDH-wildtype glioblastomas, these have been reclassified as astrocytoma, IDH-mutant, CNS WHO grade 4 in the fifth edition of the WHO Classification of Tumours of the Central Nervous System (WHO CNS5, 2021). A hallmark of WHO CNS5 is the integration of molecular markers into tumour grading. As such, IDH-mutant astrocytomas with CDKN2A/B HD are classified as grade 4 tumours independent of morphologic features. Therefore, a diagnosis of astrocytoma, IDH mutant, or CNS WHO grade 4 requires either morphologic features of a glioblastoma, namely necrosis or microvascular proliferation, or homozygous deletion of CDKN2A and/or CDKN2B (see Figure 1) [1,14].

The significance of CDKN2A/B alterations in gliomas is difficult to assess in historical cohorts. Prior to 2016, many studies classified tumours based only on morphology. Consequently, tumours previously classified as astrocytomas on morphological grounds are likely to include tumours that are currently classified as astrocytoma, IDH-mutant, oligodendroglioma IDH-mutant, 1p/19q codeleted or glioblastoma, and IDH-wildtype [15,16,17]. Interpretation of the CDKN2A/B literature is further complicated by the multiple different techniques used to interrogate the CDKN2A and CDKN2B genes (see Table 1) and whether one or both genes are interrogated. In addition, there is ambiguity concerning the significance of isolated CDKN2A or CDKN2B loss compared to loss of both CDKN2A and CDKN2B [11,13,18,19,20].

Currently, there are no consensus treatment protocols for IDH-mutant CDKN2A/B HD astrocytomas. A significant proportion (6–20%) of grade 4 IDH-mutant astrocytomas with CDKN2A/B HD were previously classified as either grade 2 or grade 3 tumours [7,11,12,13]. While some centres have started to manage IDH-mutant CDKN2A/B HD astrocytomas using the EORTC-NCIC protocol for IDH-wildtype glioblastomas, historically most of these cases would have been treated as lower-grade (Grade 2 and 3) gliomas with radiation therapy and sequential chemotherapy.

To guide understanding and management of this newly defined group, we performed a literature review on CDKN2A/B HD in astrocytomas (see Table 1 and Table 2). We present our findings in the following categories: the normal role of CDKN2A/B and effect of their deletion in translational studies; identification of CDKN2A/B deletions; CDKN2A/B deletions in clinical studies; management of tumours with CDKN2A/B homozygous deletions; and putting CDKN2A/B deletions in perspective.

**Table 1:** Timeline of the evidence landscape for *CDKN2A*/deletions in gliomas, highlighting key findings from the respective studies.

Study	Year	Importance/Findings
Rey et al. [21]	1987	Noted the glioma cell lines had an increased loss of the short arm of chromosome 9.
James et al. [22]	1991	Found that in gliomas, the loss of 9p most commonly involved the p21 region.
Serrano et al. [23]	1993	Serrano et al. then identified the gene as *CDKN2A,* which encoded the p16 protein (identified in 1993) and was located in 9p21.
Jen et al. [24]	1994	Found that *CDKN2A* deletions closely identified with *CDKN2B*
Zhang et al. [25]	1996	Mapped out the frequency of deletions occurring with *CDKN2A* deletions, including *MTAP, IFNA1,* and *IFNB1.*
Sonoda et al. [26]	1995	Demonstrated increased frequency of *CDKN2A/B* in the clinical setting.
Dehais et al. [15]	2006	One of the earliest reports of negative survival associated with *CDKN2A* homozygous deletion.
Idbaih et al. [27]	2008	Comparison of low-grade gliomas with progression to higher-grade counterparts, with loss of chromosome 9 and the *CDKN2A* locus found to be significantly associated with tumour progression.
Reis et al. [28]	2015	First to identify the prognostic role of *CDKN2A* in the setting of *IDH1/2* mutations (though they found a weak association with poor OS).
Louis et al. (WHO 2016 CNS Tumour Classification) [1]	2016	*IDH1/2* mutation status adopted into the WHO CNS Tumour Classification 2016.
Ceccarelli et al. [29]	2016	G-CIMP-low IDH-mutant astrocytomas are associated with abnormalities in *CDKN2A.*
Roy et al. [30]	2016	Investigated the impact of the loss of the 9p region in gliomas. Showed *CDKN2A* HD was not predictive in *IDH*-mutant 1p/19q non-codeleted astrocytomas but was for *IDH*-wildtype gliomas. They did demonstrate. Heterozygous loss was associated with poor OS, but mRNA expression was not altered.
Cimino et al. [19]	2017	Proposed a combined molecular model that included *CDKN2A* homozygous deletions to prognosticate in IDH-mutant astrocytomas.
Aoki et al. [31]	2018	Assessed institutional cases with validation against the TCGA database. *CDKN2A* HD alone did not correlate with survival; however, Rb pathway alterations as a group (including *CDKN2A*) were associated with poor OS.
Shirahata et al. [11]	2018	The first study to propose a grade 4 diagnosis for IDH-mutant astrocytoma in the presence of *CDKN2A/B*.
Appay et al. [12]	2019	Assessed the prognostic utility of *CDKN2A* HD, *CDK4* amplification, and *RB1* HD in *IDH*-mutant astrocytomas. Only *CDKN2A* HD predicted poor prognosis in univariate and multivariate analyses. It was also suggested that *CDKN2A* HD could define grade 4 astrocytomas.
Yoda et al. [13]	2019	Demonstrated *CDKN2A* as a strong predictor for OS in *IDH*-mutant astrocytomas.
Korshunov et al. [18]	2019	In a study of IDH-mutant ‘glioblastomas’ (now known as IDH-mutant grade 4 astrocytomas), *CDKN2A/B* HD was found to be a poor prognostic factor.
Yang et al. [10]	2020	Assessed *PDGFRA* and *CDK4* amplification, *CDKN2A* deletion, *TERT* promoter mutation, *ATRX* loss, and p53 expression in *IDH*-mutant astrocytomas. Multivariate analysis showed correlation with all three markers, and a risk stratification model was suggested using these three alterations.
Brat et al. [7]	2020	Presented the findings of the working group for grading criteria and terminologies in IDH-mutant astrocytomas in the fifth update for cIMPACT-NOW. Suggested that if CDKN2A/B homozygous deletion, necrosis, or microvascular proliferation were present, a grade 4 designation was appropriate. The term *IDH*-mutant glioblastoma would no longer exist.
Lu et al. [8]	2020	Meta-analysis of nine studies (80% low-grade and 20% grade 4) concerning the impact of *CDKN2A* HD in astrocytomas. They found *CDKN2A* HD was predictive for OS.
Satomi et al. [32]	2021	Reported a negative survival impact in *CKDN2A* HD *IDH*-mutant grade 3 astrocytomas but not in *IDH*-mutant grade 4 astrocytomas (based on morphologic criteria).
Louis et al. (WHO 2021 CNS Tumour Classification) [14]	2021	*CDKN2A/B* HD was adopted as a criterion for grade 4 IDH-mutant astrocytomas in the WHO CNS Tumour Classification 2021 (in addition to morphologic features).
Tesileanu et al. [33]	2021	Demonstrated that G-CIMP’s low methylation profile and *CDKN2A/B* HD bared an extremely poor prognosis in *IDH*-mutant astrocytomas, similar to *IDH*-wildtype glioblastomas.

## 2. Normal Role of CDKN2A/B and Effect of Their Deletion

**Figure 2:**
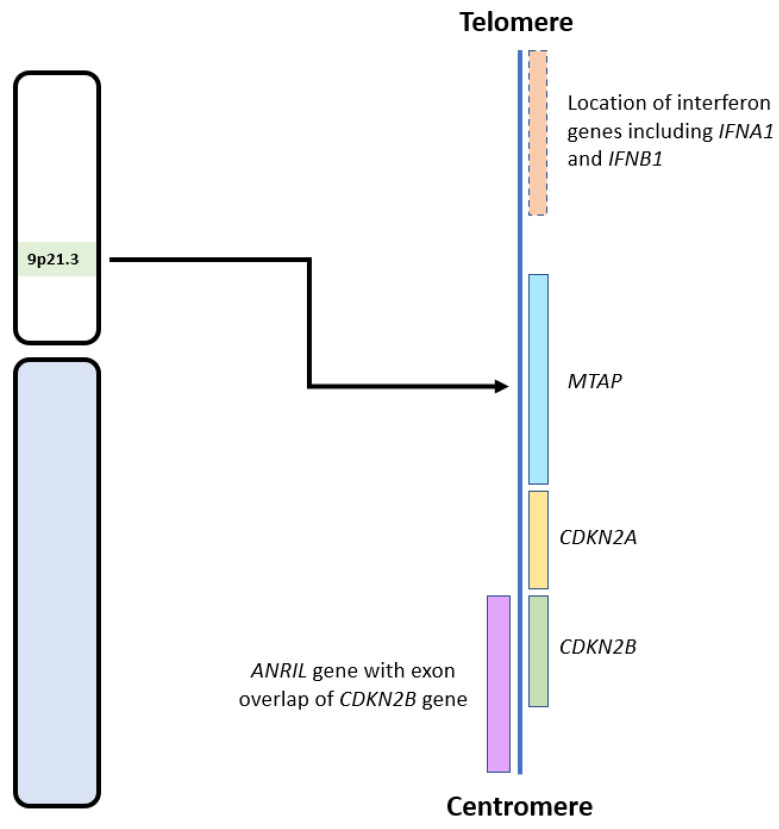
Diagram showing the locations of *CDKN2A/B* and other relevant genes on the 9p21.3 locus. Adapted from the National Institute of Health, National Library of Medicine: Genome Data Viewer [38].

*CDKN2A* and *CDKN2B* are adjacent to each other on chromosome 9p21, with *CDKN2B* being 25 kilobases (kb) centromeric to *CDKN2A* (see Figure 2) [34]. These genes code for three proteins that suppress the oncogenic cyclin-dependent kinase (CDK) pathway. *CDKN2A* encodes for p14 and p16, and *CDKN2B* encodes for p15 [35]. Early cytogenetic studies identified recurrent loss of the short arm of chromosome 9 in glioma cell lines [21,22,23,36], many involving the 9p21 locus [22,37], which includes *CDKN2A* and *CDKN2B* [23,36].

**Figure 3:**
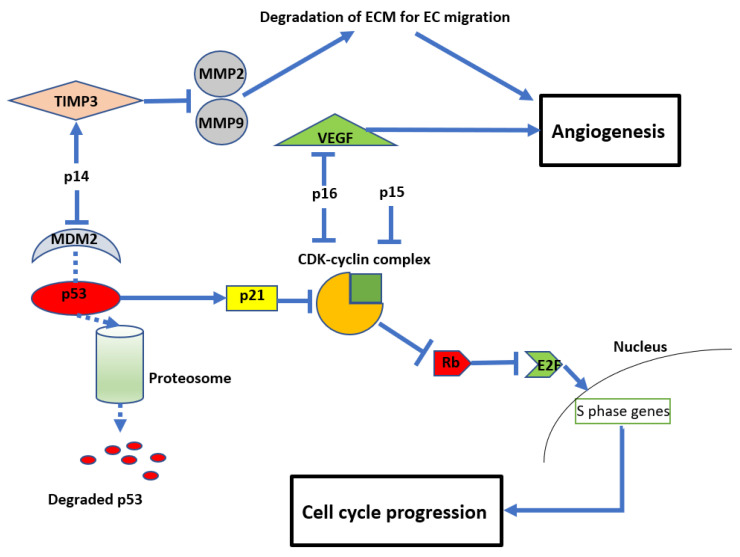
Diagram showing the anti-proliferative and anti-angiogenic effects of *CDKN2A/B* Mouse double minute 2 homolog (MDM2), tissue inhibitor of metalloproteinase 3 (TIMP3), matrix metalloproteinases (MMP), retinoblastoma protein (Rb), vascular endothelial growth factor (VEGF), and cyclin-dependent kinases (CDK).

The resultant loss of p14, p15, and p16 proteins from *CDKN2A/B* HD leads to dysregulation of the cell cycle and other parallel oncogenic processes (see Figure 2). Reflecting this, inactivation of *CDKN2A* function has been reported in a variety of other malignancies, including breast cancer, lung cancer, head and neck cancer, melanoma, and bladder cancer [9]. In normal cells, the retinoblastoma (Rb) protein prevents cell growth by binding to the transcription factor E2F, preventing its translocation into the nucleus. A complex formed by cyclin D and CDK4/6 can phosphorylate Rb, thereby releasing E2F and allowing translocation into the nucleus, leading to cell growth. The products of *CDKN2A/B*, p15 and p16, can directly inhibit the formation of the CDK4/6-cyclin D complex, maintaining the association between E2F and Rb [39,40,41] and preventing cell cycle progression. Another product of *CDKN2A* is p14, which acts on cyclin-CDK complexes indirectly by inhibiting MDM2. MDM2 tags p53, targeting it for ubiquitination and subsequent proteasomal degradation. 14 prevents MDM2 tagging, resulting in p53 stabilisation. This in turn promotes the cellular accumulation of the inhibitory protein p21, which blocks several cyclin-CDK complexes and promotes cell cycle arrest [42,43]. Due to these important functions of the protein products of *CDKN2A/B,* their deletion enhances oncogenic potential and leads to unregulated cellular proliferation (see Figure 3) [42,43].

In addition to their role in regulating cell growth, *CDKN2A/B* also impact angiogenesis (see Figure 3). For example, p14 (unrelated to its inhibition of MDM2) can also inhibit endothelial cell migration required for angiogenesis by stimulating the expression of tissue inhibitor of metalloproteinase 3 (TIMP3), which inhibits matrix metalloproteinases (MMP) 2 and 9 [44]. MMPs are required to degrade the extracellular matrix to allow endothelial cell migration and subsequent vessel formation [45,46]. Similarly, p16 inhibits angiogenesis by regulating vascular endothelial growth factor (VEGF), a well-recognised and significant biomarker in glioma development and a current therapeutic target clinically [47,48,49].

The effects of *CDKN2A/B* deletion on tumour development may also be mediated, at least in part, by co-deletion of adjacent genes in the 9p21 region (see Figure 2), such as *MTAP*, *IFNA1, IFNB1,* and *ANRIL* [25,50]. *MTAP* encodes for the protein methylthioadenosine phosphorylase (MTAP) and is located approximately 100 kb telomeric to *CDKN2A*. MTAP is required for adenosine monophosphate and methionine salvage and has a tumour suppressive effect in multiple cancers [32,51]. *IFNA1* and *IFNB1*, which encode for interferons, are also located telomeric to *CDKN2A*. Interferons are cytokines with anti-tumour effects due to their role in modulating the immune system [25,52]. Therefore, their loss also aids tumour survival and growth. *ANRIL*, an antisense long non-coding RNA (lncRNA), located centromeric to *CDKN2A,* contains *CDKN2B* within its first intron. *ANRIL* promotes pro-oncogenic gene expression and has been implicated in many malignancies, including gliomas [50]. Zhang and colleagues mapped the co-deletion of genes adjacent to *CDKN2A* in 14 cell lines, including gliomas. Only two had an isolated *CDKN2A* deletion, while the remaining ten had concurrent deletions of *MTAP* (12 cell lines), *IFNA1* (8 cell lines), and *IFNB1* (5 cell lines) (see Figure 2) [25]. Therefore, loss of the 9p21 region, inclusive of *CDKN2A/B* HD, can lead to multiple deleterious and oncogenic effects involving the loss of tumour suppressor genes and subsequent upregulation of multiple oncogenes and related pathways.

## 3. Identification of CDKN2A/B Deletions

A variety of methods can be used to evaluate *CDKN2A/B* HD. These include single-nucleotide polymorphism (SNP) microarrays [31,53], next-generation sequencing (NGS) [12,29], DNA-based methylation studies [11,13,18,19,20], and fluorescent in situ hybridisation (FISH) [10,28]. It should be noted that the accuracy of this variety of methods depends on the specific assay types used, as the genomic/cytogenetic resolution of each method differs. We are therefore unable to uniformly describe the technical parameters of each method, but have attempted to give an overview where possible. 

Although SNP arrays, NGS, and methylation arrays possess greater resolution for individual gene-level detection, many studies combine *CDKN2A* and *CDKN2B* in the assessment of HD [11,18,29,31,53]. The accuracy of these methods is determined by the degree and depth of coverage of the genes of interest. NGS methods used in the literature to date include targeted gene panels [12] and whole exome sequencing (WES) [29], whereas methylation arrays include a combination of the HumanMethylation450 (450k) and MethylationEPIC (850k) arrays (Illumina, San Diego, CA, USA) [11,13,18,19,20]

Fluorescence in situ hybridization (FISH) can be used to detect deletions and has been validated against methods utilising polymerase chain reaction (PCR) [54]. Thresholds of detection for FISH need to be around 20% to 30% tumour cells with HD [10,55]. A commonly used FISH probe in clinical diagnostic practise, the Vysis CDKN2A/CEP 9 FISH Probe Kit (Abbott Laboratories, North Chicago, IL, USA), is large and spans *CDKN2A, CDKN2B,* and *MTAP* genes [56]. Therefore, smaller deletions not involving all three of these genes may be missed. 

Immunohistochemistry (IHC) has been used to identify *CDKN2A* HD in gliomas with mixed results. Given the close proximity of the *CDKN2A* and *MTAP* genes (see Figure 2), loss of *MTAP* immunoreactivity has been suggested as a surrogate for *CDKN2A* HD [32] and has been demonstrated in mesothelioma [57,58]. In gliomas, Satomi et al. reported a sensitivity of 88% and a specificity of 98% for loss of MTAP immunoreactivity and *CDKN2A* deletion [32]. However, the authors could not demonstrate a correlation between the loss of MTAP immunoreactivity and OS in *IDH*-mutant astrocytomas [32]. 

However, Satomi et al. did show that loss of p16 immunoreactivity correlated with clinical outcome in *IDH*-mutant astrocytomas [32]. While this is supported by other studies that demonstrated p16-negative tumours on IHC had a high negative predictive value for *CDKN2A* HD in adult and paediatric morphologic glioblastomas [59], other studies reported p16/*CDKN2A* discordance with the IHC method [28]. Sensitivity and specificity for p16 immunoreactivity in detecting *CDKN2A* HD have been reported as 78–94% and 70–82%, respectively [32]. Furthermore, the full prognostic impact of *CDKN2A/B* deletions may be related to the additional loss of nearby genes, as noted above. Therefore, some tumours that are *CDKN2A/B* intact but have suppressed *CDKN2A/B* expression will have no immunoreactivity on p16 IHC but retain other genes that may confer a less aggressive phenotype. This means loss of *CDKN2A/B* in molecular studies may be more informative for prognosis than loss of p16 staining on IHC. 

## 4. *CDKN2A/B* Deletions in Clinical Studies

The advent of molecular classification introduced by the WHO 2016 CNS classification creates a distinct change in categorising gliomas [1]. Therefore, previous studies involving *CDKN2A/B* deletions need to be interpreted in the context of the WHO classification used at that time (see Table 2). We reviewed *CDKN2A/B* clinical studies in three categories: initial clinical studies, clinical outcomes of CDKN2A/B deletion in the pre-molecular classification era, and clinical outcomes of CDKN2A/B deletion in the post-molecular classification era.

### 4.1. Initial Clinical Studies 

Initial studies by Schmidt et al. [60] and Giani and Finocchiaro et al. [61] confirmed that CDKN2A HD was present in patients’ tumours and not just in glioma cell lines but did not assess *CDKN2B*. (see Table 1). Giani and Finocchiaro et al. demonstrated *CDKN2A* HD in over 30% of gliomas (not further defined) and CDKN2A HeD in 25% [61]. Moulton et al. analysed 27 glioblastomas (not further defined) and identified 9 with *CDKN2A* HD, 3 with a heterozygous deletion, and one with a point mutation [62]. 

### 4.2. Clinical Outcomes of CDKN2A/B Deletion in the Pre-Molecular Classification Era (Pre-2016 WHO CNS Tumour Classification)

Initial studies assessing the clinical impact of CDKN2A/B HD on prognosis in astrocytomas yielded conflicting results (see Table 2). This was likely due to tumour misclassification in the absence of routine assessment of IDH and 1p/19q status [63]. 

#### 4.2.1. Correlation with High- and Low-Grade Gliomas

Initial studies described the relationship between *CDKN2A/B* and biologic markers of tumour aggressiveness (tumour grade and Ki-67 index). Sonoda et al. suggested *CDKN2A/B* deletions may have a role in gliomagenesis and therefore more aggressive tumour biology. Using single-strand conformation polymorphism (SSCP) and quantitative polymerase chain reaction (qPCR), they showed an increased incidence of *CDKN2A/B* HD in high-grade gliomas (44%, n = 12/27) compared to low-grade gliomas (10%, n = 1/10) [26]. Building on this concept, Ono et al. (1996) used multiplex PCR to assess *CDKN2A/B* HD in 50 astrocytomas and found a positive correlation between the Ki-67 index and *CDKN2A* HD (5/20 grade 3 astrocytomas and 6/13 glioblastomas had *CDKN2A* HD). CDKN2A HD was not identified in 17 grade 2 astrocytomas [64]. Using SSCP and qPCR, Barker et al. (1997) analysed 42 gliomas (16 glioblastomas [5 recurrent], 15 anaplastic astrocytomas [4 recurrent], and 11 astrocytomas, oligodendrogliomas, and mixed oligoastrocytomas [1 recurrent]) and found a higher incidence of *CDKN2A* HD in higher vs. lower grade tumours (80% vs. 20%, *p* = 0.001). They also showed a similar incidence of *CDKN2A* HD in the recurrent tumours (78%) [65]. These studies confirmed that *CDKN2A* HD was associated with higher-grade tumours. 

#### 4.2.2. Correlation with Survival

In 2006, Dehais et al. reported that *CDKN2A* HD was a negative prognostic factor in a heterogeneous group of gliomas that included anaplastic astrocytomas, oligoastrocytomas, and oligodendrogliomas. Although 1p/19q status was assessed, the authors did not identify which cases had *CDKN2A* HD and 1p/19q co-deletion [15]. However, other reports did not find an association between *CKDN2A* HD and clinical outcome [66,67]. This may reflect differences in methodology and/or patient selection for tumours classified by morphology alone. One of these studies (Rich et al.) used a DNA microarray to assess the prognostic impact of *CDKN2A* deletion in patients older than 50 years. Although *IDH* status was not reported in the study, this population was likely enriched for *IDH*-wildtype tumours, and it was later shown that *CDKN2A* deletions lack prognostic impact in these tumours [66]. The other study (Zolota et al.) used p16 IHC as a surrogate for *CDKN2A* loss [67]. However, as discussed above, p16 loss is less sensitive than direct methods for assessing *CDKN2A* deletion [15].

James et al. further supported *CDKN2A* HD as a negative prognostic factor by assessing 135 gliomas for *PTEN* and *CDKN2A* copy number status. They reported an increasing frequency of *CDKN2A* deletions with grade (0% in grade 2, 14.3% in grade 3, and 27.3 in grade 4 tumours). They observed that *CDKN2A* deletions were negative prognostic indicators of survival in all 135 gliomas, but this was not seen when stratifying for grade 3 or 4 separately [16]. This lack of stratification by grade is likely related to heterogeneity within the tumour grades designated at the time, in the pre-molecular classification era. 

As interest in *CDKN2A* deletions increased, there was further focus on glioma subtypes, including oligodendrogliomas. Cairncross et al. explored the role of *CDKN2A* deletions in oligodendrogliomas using 1p/19q co-deletion instead of morphologic criteria alone. They analysed 39 morphologic oligodendrogliomas (2 of which were grade 2, the remainder grade 3). Losses involving both chromosomes 1p and 19q were strongly associated with longer overall survival, whereas *CDKN2A* deletions were independent poor prognostic factors. The authors were able to analyse 34 of the 39 samples and noted that 22 patients had 1p/19q co-deletion. It is therefore assumed that the remaining 12 cases would most likely be astrocytomas by current classification [14,17] The overall survival for *CKDN2A*-deleted gliomas was less than 2 years and occurred preferentially in gliomas without loss of 1p or 19q [17]. 

### 4.3. Clinical Outcomes in the Post-Molecular Classification Era (Post-2016 WHO CNS Tumour Classification)

#### 4.3.1. Incorporation of CDKN2A/B Status into the fifth Edition of the WHO Classification (2021)

In 2020, the Consortium to Inform Molecular and Practical Approaches to CNS Tumour Taxonomy (cIMPACT-NOW), upgrade 5, published recommendations for grading criteria and terminologies in *IDH*-mutant astrocytomas. After reviewing the literature on multiple potential prognostic biomarkers, including *CDKN2A/B* HD, other Rb pathway genes, *PIK3R1* and *PIK3CA* mutations, *PDGFRA* and *MYCN* amplification, reduced global DNA methylation, genomic instability (high copy number variants or somatic mutations), and mitotic activity and proliferation indices, they concluded that while “significant mitotic activity” should remain as a criterion for distinguishing grade 3 from grade 2 *IDH*-mutant astrocytomas, if *CDKN2A/B* HD, necrosis, or microvascular proliferation was present, a grade 4 designation was appropriate [7].

These recommendations were incorporated into the fifth edition of the WHO Classification of Tumours of the Central Nervous System 2021 [14]. While there is strong evidence to support the use of *CDKN2A/B* HD in grading *IDH*-mutant astrocytomas, several conflicting reports have been published. 

#### 4.3.2. Literature That Supports CDKN2A/B Stratification 

In 2015, Reis et al. identified *CDKN2A* deletions as a prognostic marker specifically in *IDH*-mutant grade 2 and 3 gliomas. The authors analysed 270 gliomas and identified *CDKN2A* deletions via FISH in 57/108 grade 2 astrocytomas, 31/61 grade 3 astrocytomas, 23/96 oligodendrogliomas, and 19/49 oligoastrocytomas, inclusive of both homozygous and heterozygous *CDKN2A* deletions. The authors assessed tumours for 1p/19q deletion if they were not morphologic astrocytomas and assessed all tumours for *IDH1/2* mutations by genome sequencing. They reported worse overall survival in grade 2 and 3 gliomas after adjusting for age, sex, and *IDH* mutation (HR 1.6, 95% CI = 1.0–2.4, *p* = 0.03). This significance was maintained in the astrocytoma subgroup (HR 2.0, 95% CI 1.1–3.5, *p* = 0.02) but not for oligodendrogliomas or oligoastrocytomas (HR 0.7, 95% CI 0.2–2.0, *p* = 0.5 and HR 0.8, 95% CI 0.3–2.4, *p* = 0.7, respectively). Again, a portion of these morphologic oligodendrogliomas in this cohort would no longer be classified as such without the corresponding molecularly confirmed 1p19q co-deletion. Interestingly, the presence of deletions in the *IDH*-mutant/*ATRX* expression loss astrocytoma group, without *TP53* mutation, was non-prognostic (*p* = 0.2) [68]. Furthermore, as *ATRX* loss and *TP53* mutations are strongly associated with *IDH*-mutant astrocytomas, it is unclear what this *ATRX*/*TP53* discordance represents in *IDH*-mutant gliomas. Interestingly, given the FISH probe used covers a broad genomic region at 9p21, *CDKN2B* status can be said to be assessed by proxy. This study is therefore one of the few that demonstrates the prognostic role of *CDKN2B* [28]. 

In 2016, the WHO Classification of Tumours of the Central Nervous System officially recognised that the diagnosis of oligodendrogliomas now required 1p/19q co-deletion in addition to *IDH*-mutant status [1]. While this improved prognostic accuracy for oligodendrogliomas and gliomas now known as grade 4 *IDH*-mutant astrocytomas, clinical outcomes for grade 2 and 3 *IDH*-mutant diffuse astrocytomas remained heterogenous [53,63,68,69]. 

To resolve this, Cimino et al. used The Cancer Genome Atlas (TCGA) sequencing datasets for glioblastomas, astrocytomas, and oligodendrogliomas to identify prognostic molecular markers. They found that *CDKN2A* deletions, *CDK4* amplification, and chromosome 14q loss were prognostic markers. Using these, they stratified *IDH*-mutant glioblastomas into 3 prognostically relevant molecular subgroups: M1 with chromosome 14q loss and either *CDK4* amplification or *CDKN2A* deletion; M2 with either *CDK4* amplification, *CDKN2A* deletion, or chromosome 14q loss; and M3 with no 14q loss, CDK amplification, or CDKN2A deletion. The median overall survivals for M1, 2, and 3 were 23.3, 63.0, and 94.5 months, respectively (*p* < 0.05) [19]. This was one of the first studies to suggest a combination of molecular factors for risk stratification and highlights that *CDKN2A* HD can interact with other genomic alterations [19].

These results were supported by Shirahata et al., who assessed prognostic features in 211 *IDH*-mutant astrocytoma samples. The findings were then validated using three independent cohorts of 108, 154, and 224 *IDH*-mutant astrocytomas. *CDKN2A/B* status was evaluated via the analysis of DNA-based methylation data. In the initial discovery cohort, they found 38 *CDKN2A/B* HD tumours. On univariate analysis, there was a significant negative correlation with OS and *CDKN2A/B* HD (*p* = 0.0001). They proposed three prognostic models for OS based on multivariate analysis of a discovery set and confirmation in three validation sets. In all models, tumours were classified as grade 4 if *CDKN2A/B* HD was present [11]. It is important to note that this study is the first to formally assess *CDKN2B* HD in addition to *CDKN2A* HD and is one of only three studies in the Post-Molecular Characterisation Era to include both *CDKN2A* and *CDKN2B* deletions (see Table 2). 

To compare the utility of *CDKN2A* HD and the histology-based WHO grading criteria, Appay et al. analysed 428 *IDH*-mutant astrocytomas (1p/19q non-co-deletion) and 483 anaplastic oligodendrogliomas (1p/19q co-deleted) using a combination of SNP arrays, CGH arrays, and targeted gene panel NGS data. *CDK4* amplification and *RB1* homozygous deletion were assessed in a subset of tumours. *CDKN2A* HD was associated with a dismal outcome in *IDH*-mutant astrocytomas (*p* < 0.0001 for PFS and *p* = 0.004 for OS) in both univariate and multivariate analyses. They suggested that *IDH*-mutant astrocytomas with *CDKN2A* HD should be considered grade 4 tumours irrespective of the presence of microvascular proliferation or necrosis [12]. Interestingly, *CDK4* and *RB1* alterations did not correlate with clinical outcomes.

Further reports using methylation data and FISH later emerged, indicating *CDKN2A* could stratify *IDH*-mutant astrocytomas, either alone or in combination with other molecular alterations [13,68,69]. However, the most pertinent of these latter studies was analysed by a meta-analysis assessing the association between *CDKN2A* HD and survival in *IDH*-mutant glioma [8]. The meta-analysis comprised nine studies, including 1756 (80%) LGG and 437 (20%) glioblastomas. Oligodendrogliomas and astrocytomas were defined by 1p/19q co-deletion status. Multivariate analysis identified *CDKN2A* HD as a predictor of significantly shorter PFS and OS in both LGG and glioblastoma in all included studies. Although this analysis included both astrocytomas and oligodendrogliomas, the authors noted that of the three studies reporting 1p/19q co-deletions, when the co-deletion was excluded, *CDKN2A* retained its prognostic value [8].

#### 4.3.3. Literature That Counters CDKN2A/B Stratification 

Not all studies supported the use of *CDKN2A/B* in *IDH*-mutant astrocytomas. Roy et al. analysed the 9p region lost in malignancies by analysing two cohorts (the first group being 10,985 samples from 33 different cancer types and the second group being 540 low-grade gliomas from three databases) and reported that *CDKN2A* inactivation did not promote tumour aggressiveness. Even when accounting for *IDH* and 1p/19q status (*IDH*-mutant 1p/19q non-deleted astrocytoma), there was no survival impact of CKDN2A HD. While they did show that heterozygous loss was associated with poor OS, mRNA expression was not altered. It was therefore postulated that this survival impact was due to the loss of other 9p genes [30]. It is unclear why this report differs from the majority of other studies, but it highlights that not all studies support the role of *CDKN2A/B* HD as a prognostic marker in *IDH*-mutant astrocytomas. 

Aoki et al. also failed to demonstrate the prognostic value of *CDKN2A/B* HD. They described a Japanese cohort of 308 low-grade gliomas that were comprehensively profiled for glioma-relevant genes via WES and *CDKN2A/B* via SNP array. The results were validated using a dataset of 414 LGG cases available from TCGA. Although alterations in retinoblastoma pathway genes (including *RB1*, *CDKN2A*, and *CDK4*) correlated with poor OS in general, *CDKN2A* HD alone did not correlate with OS in *IDH*-mutant astrocytomas (*p* = 0.19 for univariate and multivariate analysis) or *IDH*-wildtype astrocytomas (*p* = 0.57 for univariate analysis and *p* = 0.88 adjusted) [31].

Other studies reported a more mixed effect of *CDKN2A/B* on IDH-mutant astrocytoma prognosis. Satomi et al., using a combination of FISH and MLPA (multiplex ligation-dependent probe amplification), found a negative correlation between *CDKN2A* HD and OS in *IDH*-mutant grade 3 astrocytomas (n = 4/35 patients had a *CDKN2A* HD) (*p* < 0.001) but not in *IDH*-mutant high-grade gliomas (n = 13/27 had a *CDKN2A* HD) (*p* = 0.128). Astrocytoma was diagnosed with either the absence of 1p/19q co-deletion or loss of *ATRX* expression and strong diffuse p53 positivity. The non-significant finding in *IDH*-mutant glioblastoma and OS may be due to the small sample size [32]. Another study by Marker et al., which also employed FISH, analysed 151 *IDH*-mutant astrocytomas for *CDKN2A* HD. They reported *CDKN2A/B* as a predictor of survival in morphologically grade 4 *IDH*-astrocytomas but not for grade 2 and 3 *IDH*-mutant astrocytomas [55]. The authors of this paper suggest that this may be due to technical differences between detection methods.

## 5. Management of Tumours with CDKN2A/B Homozygous Deletions

There is no clear consensus on the treatment of *IDH*-mutant astrocytomas with *CDKN2A/B* HD, and reports related to their management are scarce. Reflecting this ambiguity, the current joint American Society of Clinical Oncology and Society of Neuro-Oncology guidelines recommend grade 4 astrocytomas be treated with concurrent temozolomide-radiotherapy with sequential temozolomide or radiotherapy alone with sequential temozolomide [70]. 

However, given the evidence that *CDKN2A/B* HD alters tumour biology (increased angiogenesis and cell growth), we cannot assume that these tumours will be as susceptible to temozolomide as their non-deleted counterparts. Unfortunately, the evidence for treatment specifically for *CDKN2A/B* HD astrocytomas is minimal. In 2000, Iwadate et al. investigated the relationship between *CDKN2A* deletion, p16 expression, and chemosensitivity to 30 different cytotoxic agents in vitro. They analysed 56 astrocytoma specimens (based on morphologic criteria, *IDH* status unknown) and found 17 specimens had p16 alterations (*CDKN2A* HD = 7, *CDKN2A* mutation = 5, p16 loss on IHC = 5). When looking at samples with p16 alterations, they found that deletions correlated with increased sensitivity to anti-metabolite agents but not to alkylating agents, antibiotics, topoisomerase inhibitors, or anti-microtubule agents [71]. 

Funakoshi et al. analysed OS in *IDH*-wildtype glioblastomas in patients before and after the use of bevacizumab. They were able to show that in the historical pre-bevacizumab group, there was a statistical difference in OS favouring the *CDKN2A* non-deleted group (OS 10.1 and 15.6 months, *p* = 0.0351). However, the significance is lost in the bevacizumab-treated group (OS 16.0 and 16.9 months, *p* = 0.1010, respectively). They proposed that the addition of bevacizumab should be considered in patients with *CDKN2A* HD glioblastoma [72]. While these studies serve as an indicator that the presence of a *CDKN2A/B* HD changes tumour response to treatment, given that they did not look at *IDH*-mutant tumours, it is hard to put them into the context of the current classification.

## 6. Putting *CDKN2A/B* Deletions into Perspective

Overall, the literature supports the use of *CDKN2A/B* homozygous deletions as a negative prognostic feature. As demonstrated in our literature summary in Table 2, most studies (n = 9/12) had a low risk of tumour classification bias, with 2 studies not showing a prognostic impact and one not describing it. However, due to the significant variance in tumour classifications used, study methodology, and techniques of deletion detection, it is difficult to appreciate the depth of this effect. It is also unclear how this molecular marker is best interpreted in the clinical context, such as the co-occurring molecular alterations or *CDKN2B* deletions in the absence of *CDKN2A* deletions. 

The clinical studies in this review span five different CNS tumour grading classifications (WHO 1993, WHO 2000, WHO 2007, WHO 2016, and WHO 2021). This means that extrapolating most of the available evidence to the current clinical situation is difficult. Varying tumour classifications have been used to describe the effect of *CDKN2A/B* HD. For example, Cimino et al. proposed different grading systems based on a combination of *CDKN2A/B* HD and other molecular markers [10,19]. However, Shirahata et al., Appay et al., and Yoda et al. proposed using *CDKN2A/B* alone as a molecular marker for grade 4 *IDH*-mutant astrocytoma, determined by a number of methods, including methylation, SNP array, and NGS [11,12,13]. Authors such as Roy et al. and Aoki et al. found that *CDKN2A/B* had no impact on survival in *IDH*-mutant astrocytomas; however, both studies used FISH [30,31]. The results of the meta-analysis by Lu et al. provide reassurance that *CDKN2A/B* HD are prognostic in *IDH*-mutant astrocytomas, given the variety of testing methods used [8]. 

The literature has not shown that *CDKN2A/B* heterozygous deletions impact survival. Roy et al. demonstrated that *CDKN2A/B* HeD did not impact survival and that survival differences seen in this group are likely due to deletions of surrounding genes [30] Therefore, the survival impact of *CDKN2A/B* deletion may be related to the loss of the surrounding genes (*MTAP, IFNA1*, *IFNB1*). 

It should be noted that point mutations were also highlighted by some of the presented research. However, the evidence for this particular alteration is very limited and does not show an impact on survival. Again, as these alterations do not result in the loss of genes neighbouring *CDKN2A/B,* this may also indicate the importance of these bystander genes in outcomes for astrocytomas with *CDKN2A/B* deletions [62,71].

There are also studies lacking a focus on *CDKN2B* deletions. In the post-molecular era, 13 studies examined *CDKN2A* deletion, with only three looking at *CDKN2B.* There is a general assumption that, due to its close proximity to and similar function to *CDKN2A*, the prognostic effect of *CDKN2B* is likely similar. Supporting this assumption are early translational studies that demonstrated that *CDKN2B* deletions, in addition to *CDKN2A,* are related to the aggressive phenotype [26]. However, this limitation still needs to be considered when interpreting the impact of *CDKN2B* HD in astrocytoma patients.

There is also a paucity of data on the management of *CDKN2A/B* HD *IDH*-mutant astrocytomas. The new entity of grade 4 astrocytoma with *CDKN2A/B* HD (WHO 2021) encompasses *IDH*-mutant astrocytomas previously treated with different regimens. Those previously classified as morphologic grade 3 astrocytomas would have been treated with radiotherapy alone followed by chemotherapy, while those previously characterised as *IDH*-mutant glioblastomas would have been treated with concurrent temozolomide-radiotherapy followed by sequential temozolomide. Therefore, which regimen should be used in *IDH*-mutant astrocytomas with *CDKN2A/B* HD is debatable. 

## 7. Conclusions

*CDKN2A/B* HD have a direct oncogenic effect through loss of cell cycle inhibition and other parallel processes and are a molecular marker that influences grading and survival in *IDH*-mutant astrocytomas. Here, we review the evidence concerning *CDKN2A/B* deletions in a historical context. Overall, the evidence supports the use of *CDKN2A/B* HD as a negative prognostic marker in *IDH*-mutant astrocytomas. However, there is a significant variation in certainty, methods used for deletion detection, and the quality of the presented literature. There are also inaccuracies resulting from misclassification of tumours in older studies based on the revised WHO classification. These limitations hamper conclusions regarding the certainty and depth of impact *CDKN2A/B* HD has on prognosis and management and how this impact is affected by other co-occurring molecular alterations. Therefore, the strongest evidence for *CDKN2A/B* HD in *IDH*-mutant astrocytomas must come from prospective reports with the current WHO 2021 classification. Furthermore, clinical trials that use the current WHO 2021 classification are required to determine the optimal management of *IDH*-mutant astrocytomas with *CDKN2A/B* HD.

## Figures and Tables

**Table 2:** Summary of clinical studies addressing the prognostic impact of *CDKN2A/B* HD. The classification used is described as pre-molecular classification (pre-WHO 2016) or post-molecular classification (post-WHO 2016). Bias resulting from poor translatability of glioma classification to current classification is ranked as “Low”, “Medium”, or “High”. The bias ranking was performed qualitatively by author consensus.

Study	Year	Design	Sample Size (n) *	Method CDKN2A/B Assessment	Publication Relative to Revised WHO	Risk of Misclassification Bias	CDKN2B Assessed?	Prognostic Impact
*Rey* et al. [21]	1987	Retrospective analysis	34	Cytogenetic testing	Pre-WHO 2016	High	NA—identified 9p loss only	Not described
*James* et al. [22]	1991	Retrospective analysis	20	RFLP analysis	Pre-WHO 2016	High	NA—identified 9p loss only	Not described
*Sonoda* et al. [26]	1995	Prospective analysis	37	PCR-SSCP	Pre-WHO 2016	High	Yes	Not described
*Dehais* et al. [15]	2006	Retrospective analysis	156	LOH testing	Pre-WHO 2016	High	No	Negative impact
*Idbaih* et al. [27]	2008	Retrospective analysis	16	CGH array	Pre-WHO 2016	High	No	Negative impact
*Reis* et al. [28]	2015	Retrospective analysis	270	FISH	Pre-WHO 2016	Moderate	Yes **	Negative impact
*Ceccarelli* et al. [29]	2016	Retrospective analysis	820	NGS, SNP array	Pre-WHO 2016	Low	No	Not described
*Cimino* et al. [19]	2017	Retrospective analysis	1062	Two separate databases accessed—TCGA (NGS); and German glioma network (DNA-based methylation analysis)	Post-WHO 2016	Low	No	Negative impact
*Shirahata* et al. [11]	2018	Retrospective analysis	697	DNA-based methylation analysis	Post-WHO 2016	Low	Yes	Negative impact
*Appay* et al. [12]	2019	Retrospective analysis	468	SNP array, CGH array, NGS	Post-WHO 2016	Low	No	Negative impact
*Yoda* et al. [13]	2019	Retrospective analysis	178	DNA-based methylation analysis	Post-WHO 2016	Low	No	Negative impact
*Korshunov* et al. [18]	2019	Retrospective	97	DNA-based methylation analysis	Post-WHO 2016	Low	Yes- but only addressed grade 4 astrocytomas	Negative impact
*Yang* et al. [10]	2020	Retrospective	160	FISH	Post-WHO 2016	Low	No	Negative impact
*Lu* et al. [8]	2020	Meta-analysis	2193	NA	Post-WHO 2016	Low	No	Negative impact
*Satomi* et al. [32]	2021	Retrospective	178	MLPA and/or FISH to validate MTAP IHC	Post-WHO 2016	Low	No	Negative impact for grade 3 *IDH*-mutant astrocytoma but not grade 4
*Tesileanu* et al. [33]	2021	Retrospective analysis	654	DNA-based methylation analysis	Post -WHO 2016	Low	Yes	Negative impact
*Roy* et al. [30]	2016	Retrospective analysis	540	DNA sequencing	Pre-WHO 2016	Low	No	No impact
*Aoki* et al. [31]	2018	Retrospective analysis	722	WES, SNP array	Post-WHO 2016	Low	No	No impact ***

* Note that n includes all gliomas addressed in the study, and if validation cohorts were used in the study, this is included in the total sample size number. ** Note that while Reis et al. did not officially investigate *CDKN2B* loss, given the large FISH probe used, *CDKN2B* would have been included in the FISH analysis [28]. *** *CDKN2A* HD did not have an impact on OS on univariate analysis, but analysis using all genes in the Rb pathway was significant. Abbreviations: not applicable (NA); polymerase chain reaction-single-strand conformation polymorphism (PCR-SSCP); fluorescence in situ hybridization (FISH); restriction fragment length polymorphism analysis (RFLP analysis); single-nucleotide polymorphism (SNP); multiplex ligation-dependent probe amplification (MLPA); immunohistochemistry (IHC); whole exome sequencing (WES).

## Data Availability

No new data were created or analysed in this study. Data sharing is not applicable to this article.
